# Field methods for above and belowground biomass estimation in plantation forests

**DOI:** 10.1016/j.mex.2020.101192

**Published:** 2020-12-19

**Authors:** Trinh Huynh, David J. Lee, Grahame Applegate, Tom Lewis

**Affiliations:** a Forest Research Institute, University of the Sunshine Coast, Maroochydore Dc, Queensland, Australia; b Forest Science Institute of Central Highlands and South of Central, Dalat, Vietnam; c Department of Agriculture and Fisheries, Gympie, Queensland, Australia

**Keywords:** Destructively sampled trees, Root systems, Wood density, Canopy area

## Abstract

A practical and cost-effective destructive sampling method for estimating above and belowground biomass of *Corymbia citriodora* subspecies *variegata* grown in plantations is described. The methodology includes details on selecting sample trees, weighing tree components in the field, excavating root systems and obtaining fresh weights and laboratory analyses of components to determine oven dry weights. The development of these sampling procedures is a basic step towards successful, consistent collection of biomass data in 18-20 years old plantation forests. This methodology was developed for eucalypt hardwood timber plantations in Queensland, Australia. However, these procedures can be applied to plantations elsewhere as well as to trees in native forest environments with minor modifications. The methodology developed for field sampling of the tree components and the derivation of allometric relationships for predicting individual tree biomass (above and belowground) highlighted the following:

• Accurate quantification of above and belowground biomass of eucalypts.
• Description of measured variables for developing allometric relationships.
• Integration of field and laboratory measurements to streamline data collection.

## Graphical abstract

**Figure:**
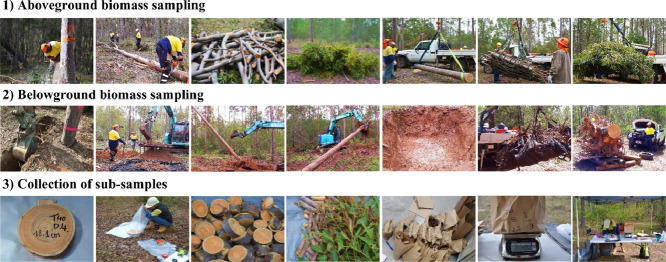

**Specifications table**

**Table:** 

Subject Area	Environmental Science
More specific subject area	*Tree biomass and carbon stocks*
Method name	Biomass and carbon accumulation in eucalypt plantations
Name and reference of original method	• IPCC Methodological Guidelines (Source: IPCC. 2006. Guidelines for National Greenhouse Gas Inventories**,** 1-66, Volume 4: Agriculture, forestry and other land use. • National Carbon Accounting System Technical Report no. 47. (Source: Ximenes, F., Gardner, W. & Marchant, J. 2005. Total biomass measurement and recovery of biomass in log products in spotted gum (*Corymbia maculata*) forests of SE NSW. National Carbon Accounting System Technical Report no. 47.
**Resource availability**	N/A

## Method details

### Background

Woody ecosystems provide the capacity for sequestering carbon and offsetting carbon emissions [1]. Woody ecosystems, including well managed plantation forests with adequately resourced fire management prevention strategies therefore play an important role in reducing global greenhouse gas emissions through storing carbon in various pools [2]. In Australia, previous research has identified the important role that production forests can play in greenhouse gas mitigation, both through storage of carbon and by providing society with low emission products [3,4].

The Intergovernmental Panel on Climate Change (IPCC) produced assessment reports, technical papers, methodologies and other technical guidelines that have become fundamental references which are now widely applied by policymakers, scientists and other experts to estimate biomass and forest carbon worldwide [1]. These guidelines have been improved and updated since 1990 with detailed conditions for each country [5]. There are also numerous methods for estimating biomass and forest carbon presented in technical reports since 1999 specifically for Australian forests [6], [7], [8]. Whilst a wide range of different methodologies have been used for determining tree biomass in native forests, a consistent methodology for above and belowground biomass estimates in commercial plantations is less well developed and have focused on temperate eucalypts [9].

Forest biomass can be estimated using non-destructive and destructive sampling methods. It is often impractical to destructively sample all standing trees to estimate biomass due to the negative environmental impacts, wastefulness, and high cost of data collection [10]. Determining generalized allometric equations to assist in rapid field biomass estimates and remote sensing techniques are commonly applied to estimate forest biomass at larger scales [11]. Developing allometric equations for specific forest types based on destructively sampled trees to ensure more accurate and reliable estimates at a local scale is therefore needed [8]. Weighing tree components is considered one of the most reliable approaches for estimating biomass of trees across a range of diameter classes [11]. Destructive sampling and biomass studies are undertaken by dividing the chosen sample trees into their components (e.g. stem, branches, foliage and roots) [1].

Previous research has established relationships between the weight of tree components and independent variables such as: diameter at breast height (DBH), tree height (H) [1,^10^,[12], [13], [14], [15], [16], [17], [18], [19], [20], [21], canopy area (CA), and wood density (WD) [13,^14^,16]. Developing the best relationships for the prediction of aboveground biomass (AGB) and belowground biomass (BGB) often involves one or more of these variables [22].

### Selection of trees to sample

When developing regression equations using destructively sampled trees, selection of appropriate sample trees across the range of tree diameters is important to minimize the bias of biomass estimates. There are several methods used to select sample trees, including selection of dominant trees in a stand or the largest, average and smallest individuals [12].

A general principle to keep in mind is that the more trees sampled, the smaller the standard error for biomass equations [13]. In addition, large trees are more important to sample than the smaller trees as the major proportion of forest biomass is represented by these trees [10]. However, the number of sample trees selected also depends on funding and the purpose of study. In some cases, time and cost determine the sample size, instead of considerations related to the precision of the predictions [14]. Sampling in timber plantations generally requires fewer sample trees than in natural forests as they usually have smaller variation in tree size, and habit for a given age, and they are generally represented by a single species with only one stem with a common planting date. Based on modelling, sampling between 17 and 95 trees were found to be suitable to predict woody biomass with a standard deviation within 5% of the mean for the best performing stem diameter selection algorithm [23].

**Fig. 1:**
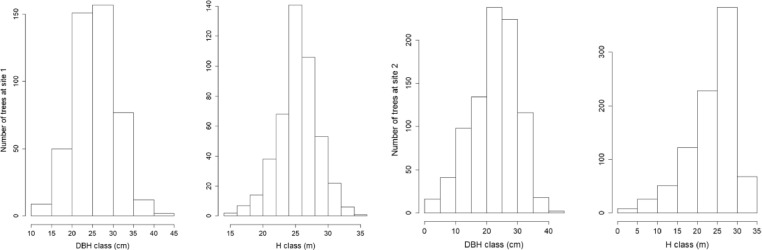
Distribution of diameter (DBH) and height (H) at site 1 (left) and site 2 (right).

The selection of sample trees should ensure that the full range of tree sizes is sampled, by dividing the trees in the stand into DBH classes. At the sites used in this study, pre-sampling surveys determined that trees ranged in diameter from 15.0 to 45.0 cm DBH (site 1) and from 3.0 to 42.0 cm DBH (site 2). The distribution of DBH and height (H) classes at the two sites are shown in Fig. 1. Trees included in this study were divided into six DBH classes: (1) 15–20 cm; (2) 20.1–25 cm); (3) 25.1–30 cm; (4) 30.1–35 cm; (5) 35.1–40 cm; (6) 40.1–45 cm, with trees selected in each DBH class.

We found the trees under 15 cm DBH were unsuitable for inclusion, as they were less frequent and were suppressed with many defects. In our case, more trees were sampled for AGB than for BGB, due to the additional costs associated with the excavation of the belowground components in order to accurately determine BGB. We aimed to sample a minimum of two trees in each diameter class for BGB determination, but time and budget constraints meant we could sample a total of 11 sample trees. Forty individual trees were sampled for AGB determination. In addition, tree selection focused on trees that were representative of the stand. In this study, these were healthy trees, with single stems.

### Field preparations prior to felling trees

**Fig. 2:**
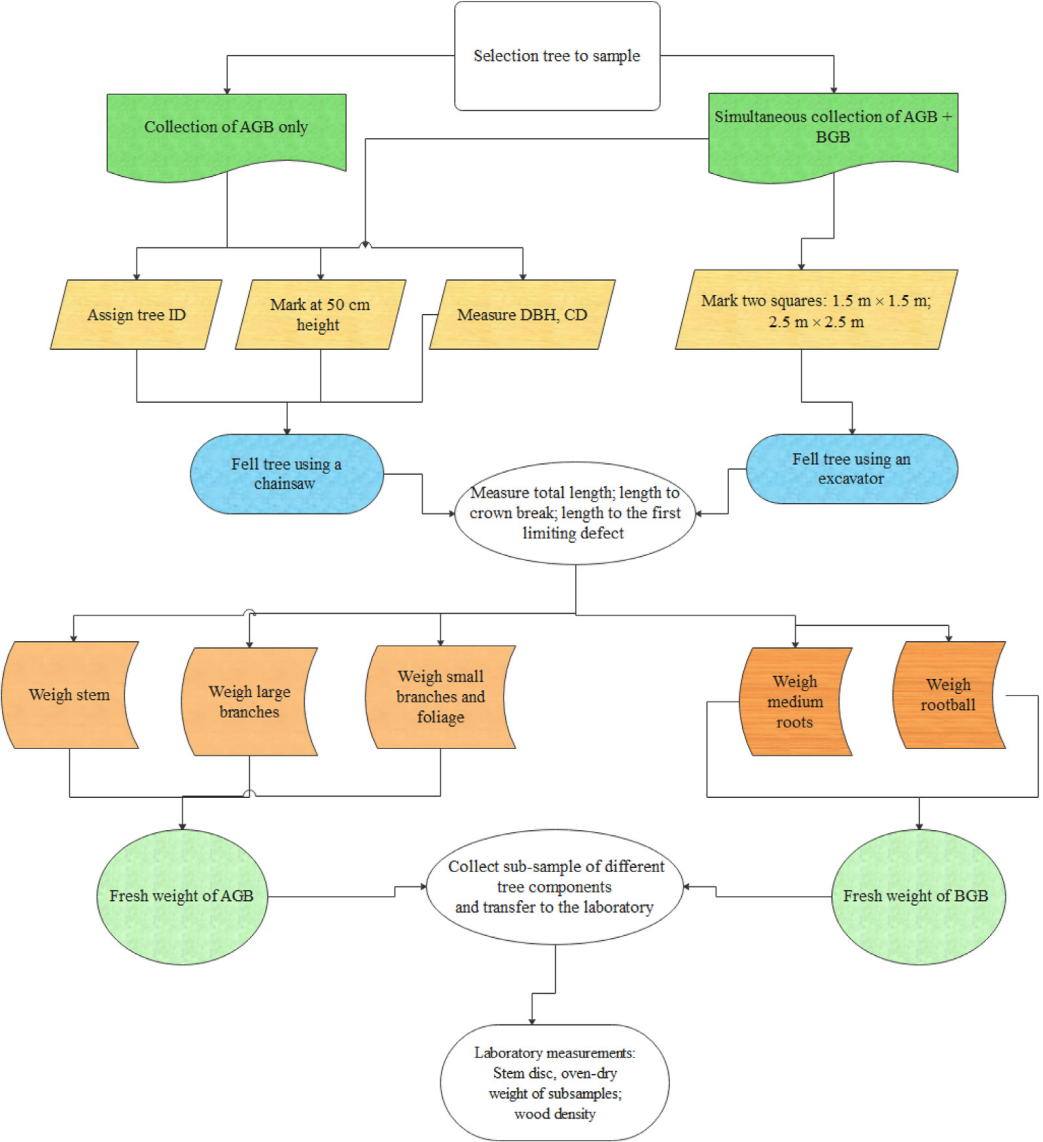
Diagram of fundamental steps to collect AGB and BGB in the field.

Prior to felling sample trees, a number of steps (Fig. 2) were undertaken to ensure the sample trees were sampled correctly as follows:

• Each tree to be sampled was assigned a tree identification number (ID). The area around the selected tree was cleared of existing vegetation to avoid contamination with non-sample material. This can be done efficiently using an excavator or chainsaw.
• Sample trees were marked at 50 cm height from the ground. This height facilitated tree felling. This position also marked the boundary between the aboveground components and the belowground components for this study. A 50 cm stump also facilitated removal of the rootball by the excavator, enabling the grapple on the excavator to securely hold the stump to remove it from the ground and shake it to remove the loose soil (as detailed in the BGB sampling section below).
• Diameter at breast height (DBH) in cm was recorded on all sample trees. Crown diameter (CD) m was measured in two perpendicular directions; along the planting row and across the planting row for each sample tree. Average crown diameter and canopy area (CA, m^2^) was then calculated using Eq. (1).
  (1) $$\text{CA}=\frac{П.\;CD^{2}}{4}\text{where},\;\text{CD}\;\text{is}\;\text{average}\;\text{crown}\;\text{diameter}\;\left(m\right)$$

### Preparation prior to weighing tree components

Although simple in principle, weighing large trees requires logistical planning and access to machinery such as chainsaws, excavator, large sieves and large load cells. An accurate field spring balance is one of the most important pieces of equipment to accurately record the weights of lighter tree components. This study used three different types of digital scales in the field and two precision balances in the laboratory as detailed in Table 1.

**Table 1:** Description of different scales and balances used in the field and laboratory.

Name	Weight capacity	Description
Cattle scale with digital display	2000 kg (0.2 kg precision)	Display in kilogram. This scale involves two load beams and a monitor powered via 12V car battery. To install the scale, a flat piece of plywood was placed on the levelled load beams and car tyres were placed on plywood to cushion the weight of the samples. The tared cattle scale was mainly used to weigh rootballs as it's fixed plate design meant samples had to be moved to it rather than it to the sample.
Digital crane scale	500 kg (0.02 kg precision	Display in kilogram. This scale is suitable to measure the weight of the samples when suspended. It attached to a Hiab crane mounted on a vehicle. Samples are lifted in a cargo net or in tared lifting straps. This system was used to weigh sections of the tree: stems, large branches, small branches and foliage along with smaller rootballs and small roots.
Digital balance	10 kg (0.005 kg precision)	Display in kilogram. The scale was placed on a table top to ensure it remained level to prevent errors when weighing sub-samples from the different components of the trees (described below). The accuracy of the balance can be affected by wind and sunlight, therefore the balance needs to be placed in a tent and kept out of the wind.
Laboratory balance	10 kg (0.1 g precision) and 40 kg (0.1 g precision)	Display in gram. The balances were placed on a tabletop in the laboratory. The 10 kg balance was used to weigh dry-weight samples and the 40 kg balance was used to measure wood density. These balances were calibrated to minimise errors.

### Sampling aboveground tree components (AGB)

Trees were felled either using a chainsaw or excavator. The chainsaw was used to fell trees selected for collecting only AGB data. For those trees used to determine both AGB and BGB data, an excavator was used to push the whole tree onto the ground (after excavating trenches around the rootball, see details on BGB sampling) prior to cross-cutting the stem at the 50 cm above ground level mark.

After the tree was felled, a 50 m tape was used to measure total tree length (height) in metres. Length of the bole to the crown break was recorded as bole height (HC). The crown break was defined as the location where the first major branches of the crown, connect to the bole of the tree. Length of bole to the first limiting defect was also recorded as defect height (HD) to determine the potentially merchantable section of the stem (summary data presented in Table 2).

**Table 2:** Descriptive statistics of the data collected at two sites.

Descriptive data	Min	Mean	Max
DBH (cm)	17.1	28.2	42.0
H (m)	20.2	26.2	32.0
CD (m)	2.7	5.8	9.8
HD (m)	6.8	13.5	22.0
HC (m)	9.0	13.9	20.6

DBH, H, CD, HD and HC are diameter at breast height (1.3m), tree height, crown diameter, height to the first limiting defect and height to crown break, respectively.

**Fig. 3:**
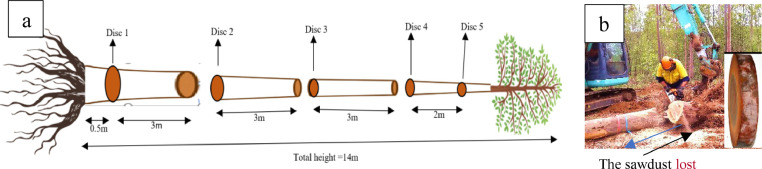
(a) Stem sections used to measure stem biomass and location of disc samples. The first disc was cut at the bottom of first log, the last disc was cut at the first limiting defect, (b) the weight of the sawdust resulting from the cutting the discs was estimated for each stem section.

The fresh weight of all aboveground tree components was then obtained to nearest 0.1 kg, as follows:

Stem: The stem was divided and marked with coloured paint into 3.0 m lengths from the base of bole (50 cm above ground level) to the height of the first limiting defect and cross cut into 3.0 m sections. Discs of 40 mm width were cut at the bottom of each section using a chainsaw with the last disc taken at the position of the limiting defect as shown in the Fig. 3a. Between five and eight discs were collected for each tree, as potentially merchantable stems were generally between 12.5 m and 20 m.

There were 223 discs collected from 40 sample trees. These discs were weighed using a 10 kg digital balance and later added to the total stem weight. The average width of chainsaw cuts was determined to estimate the loss of sawdust (Fig. 3b). In this case the average width of the cut was 8 mm (i.e. 16 mm for each disc). The formulae used to estimate sawdust lost for each disc is described in the laboratory measurements section.

**Fig. 4:**
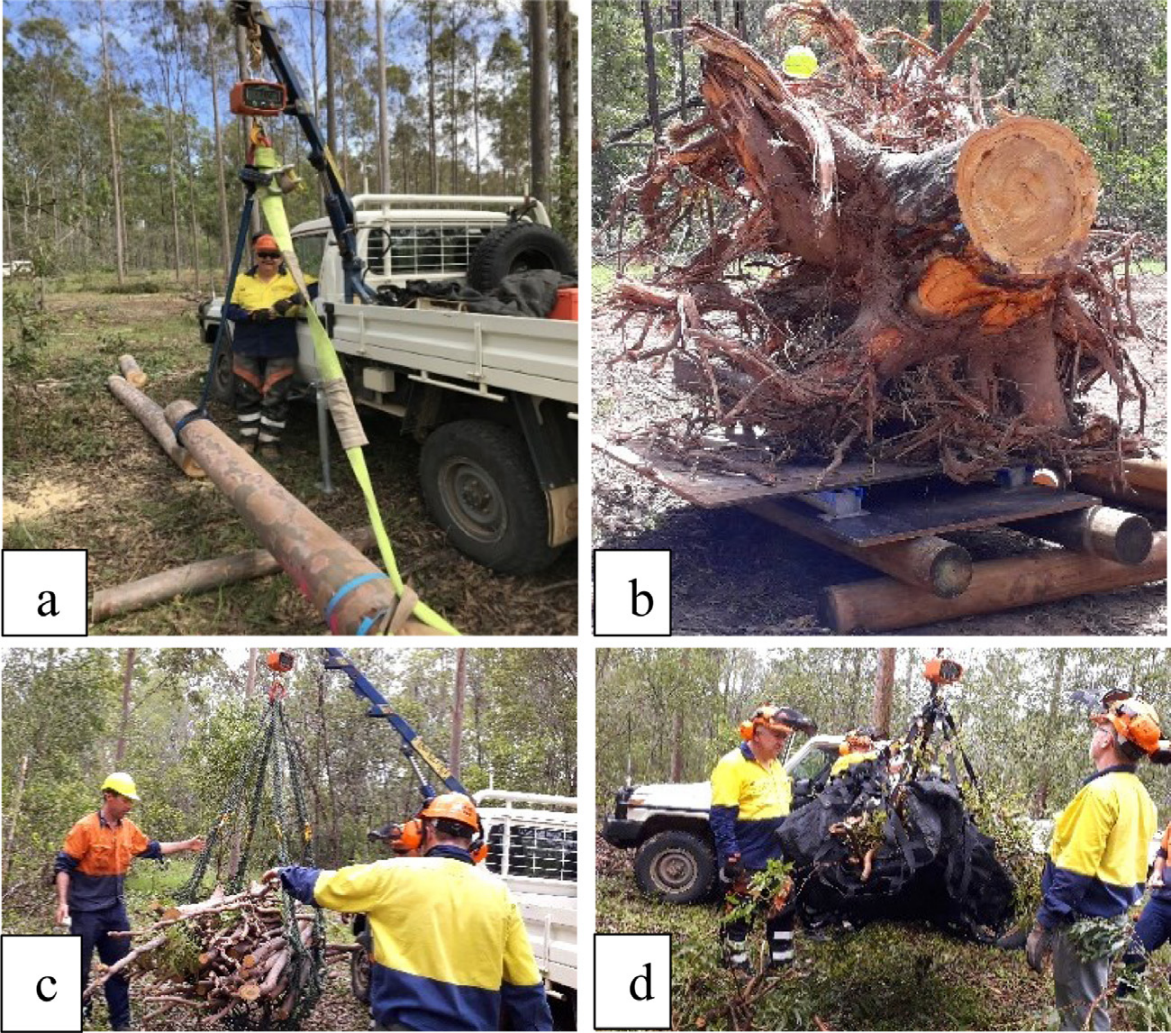
Weighing tree components in the field to obtain fresh weight: (a) 3.0 m section of the main stem measured using digital crane scales lifted using a Hiab, (b) rootball measured on cattle scales, (c) large branches, and (d) small branches and foliage lifted in a cargo net using the Hiab and digital crane scale.

Large stem sections (diameter > 25 cm) were weighed using the tared cattle scale (load cells) or the digital crane scales. Small sections (diameter < 25 cm) also 3 m in length were lifted in a cargo net or into lifting straps to facilitate weighing using the digital crane scale as are shown on Fig 4a.

Crown: Biomass of the crown includes all branches and foliage. Foliage accounts for only up to 1-2% of the AGB in mature native eucalypt forests [12] and 3-5% across a range of *Eucalyptus* species in a northern Australian savanna [24,25]. We therefore combined foliage, small branches (<2 cm diameter), buds, capsules or flowers into one component for this study. The crown component was recorded as ‘small branches’ (SB, kg), large branches (LB, kg) down to a minimum diameter of 2 cm and recorded separately.

Depending on the objectives of the sampling, total crown weight could be determined without separating small and large branches, but adequate sub-sampling of both components is important to accurately determinate moisture content of each component. These two sub-components were separated after cutting branches with a chainsaw and large loppers. Large branches were placed in a cargo net and weighed using a digital crane scale lifted on a Hiab (Fig. 4c). Small branches were weighed in a similar manner (Fig. 4d).

In order to determine the moisture content of various tree components, a sub-sample was taken and weighed in the field using a 10 kg electronic balance with an accuracy of 5 gram, as follows:

*Stem sub-samples:* The diameter of each disc removed at 3 m intervals was measured using a diameter tape. Discs were labelled (both on the discs and the heavy-duty plastic bags they were stored in) using a permanent marker as D_01_, D_02_ and D_0i_ respectively (i refers to i^th^ disc). Plastic bags were used to reduce moisture loss during transportation to the laboratory where they were kept in a -20°C freezer until they could be weighed and processed as described in the section on laboratory measurements.

*Crown sub-samples:* At least 2 kg sub-samples of the LB and SB were taken for each tree. After weighing and recording fresh weights these samples were placed into paper bags. The sub-samples were taken randomly from different positions of the crown. For example, small branches were collected from throughout the tree crown. All sub-samples were carefully labelled for identification in the laboratory (e.g. T1-LB = Tree 1 – large branches and T1-SB = Tree 1 – small branches). Sub-samples were transferred to the laboratory for oven-dry weight determination.

### Sampling belowground tree components (BGB)

The area to be excavated was identified using coloured paint to guide the operator of an 8-tonne excavator with a 45 cm wide bucket. A square 1.5 × 1.5 m was marked around the tree stump with the tree in the centre of this square.

**Fig. 5:**
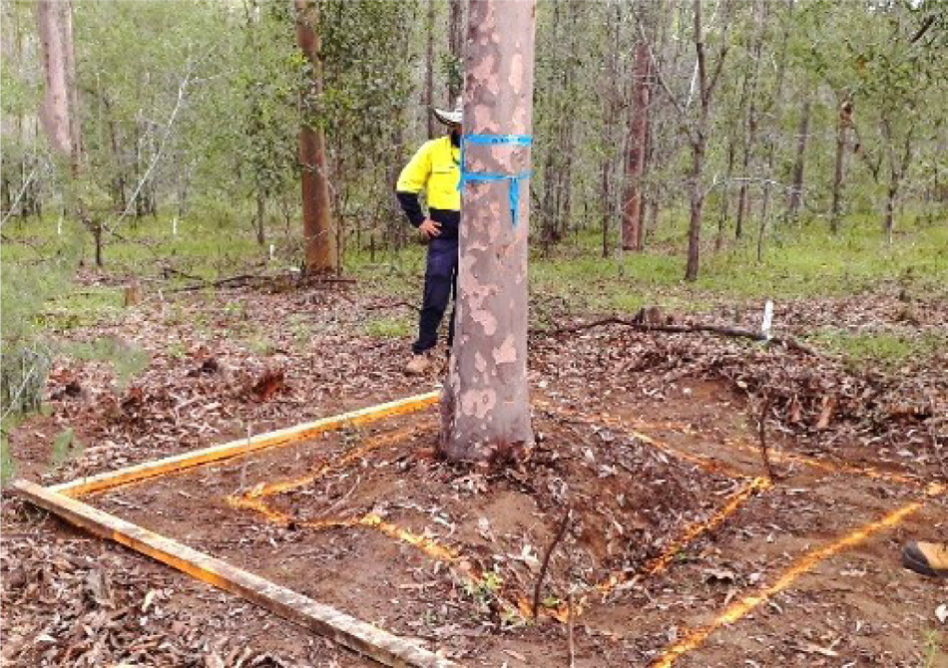
Painted excavated area to define the limits of the excavation and the rootball monolith (1.5 m × 1.5 m × 1.0 m deep; smaller square) containing the majority of the roots. Area around rootball monolith (larger square was 2.5 m × 2.5 m) was dug by an excavator to 1.0 m depth. Any roots in this outer area were collected, before sampling the rootball monolith.

An additional square was marked around the outside of area of the rootball to create a 2.5 × 2.5 m square. The rootball was classified as all biomass from the stump height marked at 50 cm from the ground, to a depth of 1.0 m below the surface and enclosed by an area marked as 1.5 × 1.5 m (Fig. 5).

**Fig. 6:**
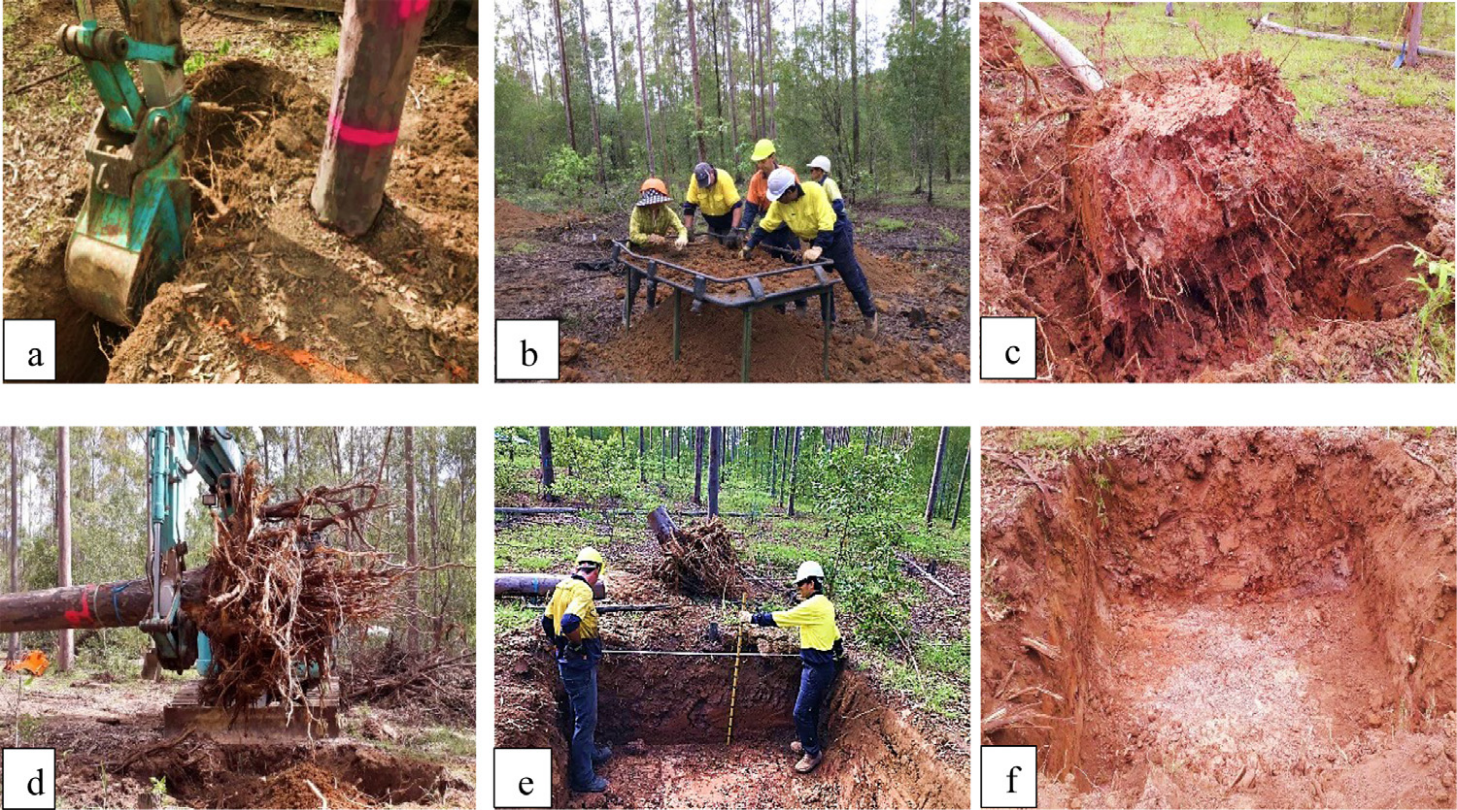
Steps for root excavation for BGB: (a) a trench 0.5 m wide was dug around the rootball monolith of the tree, (b) soil sieved to collect medium roots from the trench, (c) and (d) excavator pushed the tree over and removed soil from the rootball, (e) height sticks and measuring tapes were used to measure the BGB hole dimensions, and (f) 2.5 m × 2.5 m × 1.0 m depth hole with all roots removed from the excavated area.

The rootball and all medium roots (>2 mm diameter) of individual trees were sampled. An excavator with a 45 cm bucket dug a trench 0.5 m × 1 m deep around the outside of rootball (Fig. 6a) and soil from this trench was placed on a sieving table (a steel mesh frame) with a 4 cm × 4 cm mesh to collect any roots (> 2 mm) (Fig. 6b). After digging four trenches to 1 m depth × 2.5 m long, the excavator pushed the tree over and removed the intact rootball (Fig. 6c and Fig. 6d). The stem, large and small branches was severed from the stump with a chainsaw at 0.5 m and processed as described above for AGB.

**Fig. 7:**
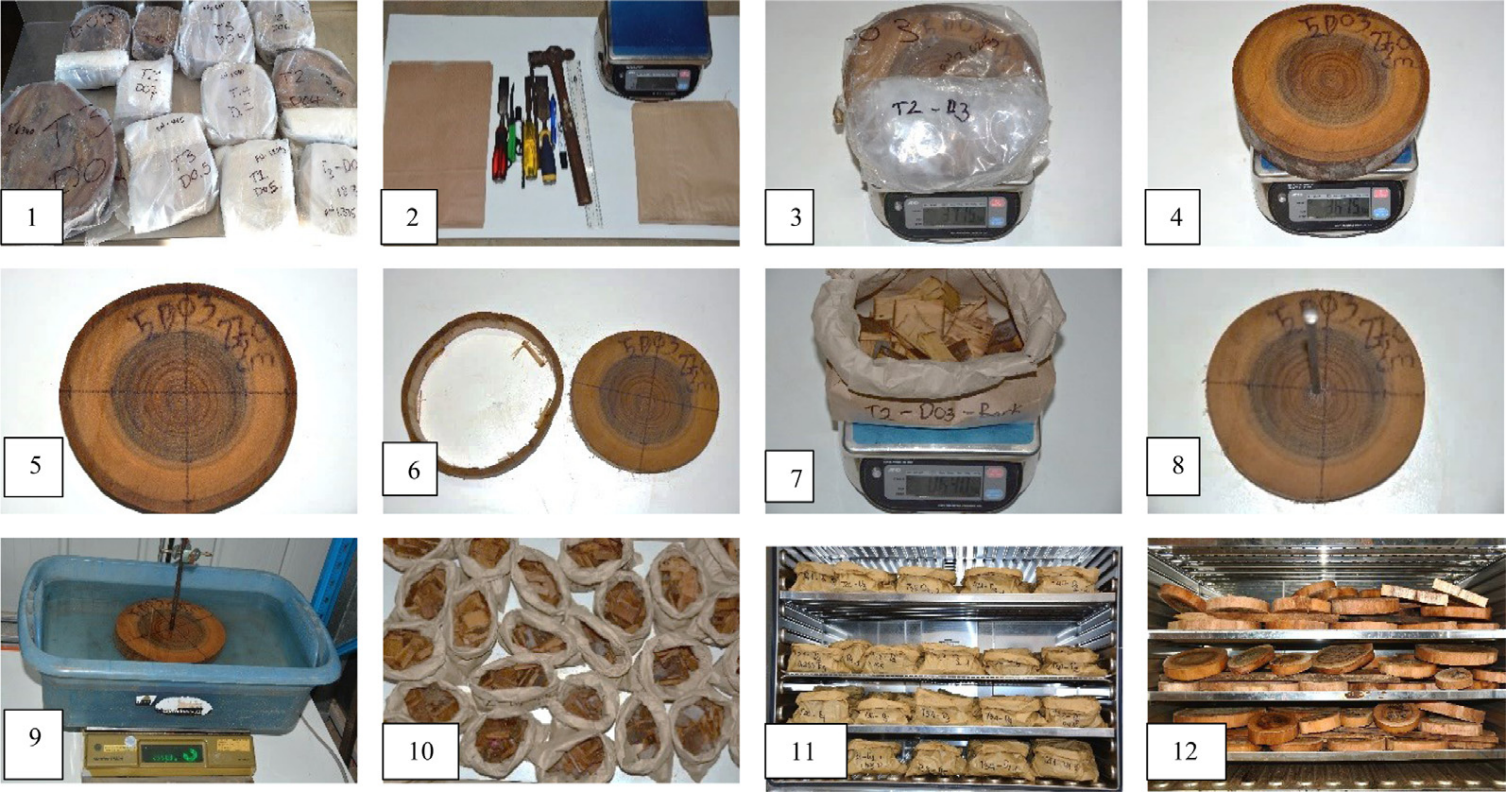
Steps for measurement of stem discs in the laboratory: (1) defrosting discs, (2) tools to sample disc, (3) weighing disc inside the plastic bag, (4) re-weighing disc without plastic bag, (5) measuring bark thickness, sapwood and heartwood, (6) bark removed from the disc, (7) weighing fresh bark, (8 and 9) immersing disc for measurement wood density, (10 and 11) bark samples split into small pieces before being placed in paper bags and drying in the oven, and (12) drying discs in the oven.

The excavator lifted the rootball and shook the soil from it. Dropping the rootball into the excavation hole also helped removed the soil. The broken roots were collected from the excavation hole and identified as a part of the rootball. The rootball was then removed from the excavated area. For trees growing in soils with a high clay content, a small crowbar was used to remove soil stuck to the rootball taking care to collect any broken and discarded roots during this process.

The excavator then removed the remaining soil in the 2.5 m × 2.5 m × 1.0 m hole and placed it on the sieving table so all of the roots in the pit could be collected free from soil. Once the soil was completely removed from the hole, pruning shears or loppers were used to harvest roots remaining inside the area. These roots were weighed as part of the medium roots.

The dimensions of the excavation area were measured using graduated height sticks and measuring tapes (height, lengths of sides and top diagonals) to allow a more precise calculation of ratio of root to soil in the excavated area (Fig. 6e).

Excavation for the belowground biomass was destructive, damaging the soil at the sample sites. This study was necessary so we can limit the amount of soil damage done in other plantations, by developing non-destructive methods to accurately estimating BGB and AGB, based on the data collected. To minimise the damage at the site the excavator backfilled the hole with the sieved soil.

The sieving process took approximately 2 hours with eight staff, but varied depending on the clay content of the soil. In our case, it took longer to collect medium roots in heavy-textured soils in comparison with trees grown on sandy soils with less clay. The next part of the process involved obtaining fresh weight of the roots. The rootball was weighed using cattle scales (Fig. 4b) and the medium roots were weighed using a digital crane scale lifted using the Hiab.

Sub-samples of the rootball and medium roots (over 2 kg per tree) were taken for oven-dry weight determination and labelled using a similar system to that used for the AGB components.

### Laboratory measurements

#### Dry sub-samples

The sub-samples of all components except for stem discs were cut into small pieces and placed in paper bags that were not packed tightly, to assist the drying process and dried until constant weigh was achieved in forced draft ovens. Foliage, small branches and medium roots were dried at a temperature of 65-70 °C and took at least 2-3 weeks to dry. The other samples (stem discs, rootball and large branches) were dried at a temperature of 100-105 °C [21] and took at least 4-5 weeks to reach constant weight. Sub-samples were weighed immediately after removal from the oven, in a closed room as samples take up moisture quite rapidly.

Oven-dry weight (Od wt) of rootball, medium roots, large branches and small branches was used to determine moisture content to convert to dry weight of each tree component (Eq. 2).

(2) $$\text{Dry}\;\text{weight}=\frac{\text{Od}\;\text{wt}\;\text{of}\;\text{subsample}}{\text{Fresh}\;\text{wt}\;\text{of}\;\text{subsample}}\;\times \text{Fresh}\;\text{wt}\;\text{of}\;\text{component}\;$$

### Stem disc measurements

The discs used for estimation of dry stem biomass were also used for estimation of wood density. Frozen discs were left overnight to defrost and kept inside the plastic bags. Discs were then removed from plastic bags and thickness of the bark, sapwood and heartwood measured. The sapwood content (%) of each section was calculated as the average sapwood content of the butt and top discs [26] (Eq. 3). Oven dry weight of discs were also used to determine moisture content and dry weight of bole using Eq. 2.

(3) $$\begin{array}{ccc} \text{Sapwood}\;\text{content}\left(\%\right) & = & (\text{Average}\text{disc}\text{area}\left(cm^{2}\right)-\\ & & -\;\text{Average}\;\text{heartwood}\;\text{area}\left(cm^{2}\right))/\text{Average}\;\text{disc}\;\text{area}\left(cm^{2}\right)\times 100 \end{array}$$

### Wood density measurement

The bark was detached from the disc using a hammer to remove the whole ring and weighed on a balance. Chisel used to split the stem bark into small pieces before oven drying. This component was then used to estimate stem bark. The stem disc (without bark) was weighed and fresh weight recorded (g ± 0.1). The volume of stem disc (bark off) was then determined (cm^3^ ± 0.5 cm^3^) using the Archimedean water displacement method, as follows (Fig. 7:

Approximately 20 litres of water was poured into a container, with a small amount of a wetting agent such as Teepol added to reduce air bubbles. The container was placed on a balance and tared prior to immersing the disc.

The stem disc was just fully submerged in the container, avoiding touching the sides of the container. After the balance stabilised, the weight was recorded as green volume (cm^3^).

After the disc was removed from the container, stem bark and stem disc were placed in a forced draft oven at 75 °C for bark samples and 100-105 °C for wood discs until constant weight was attained. Wood density (WD) of each disc was calculated (Eq. 4) and WD of each sample tree estimated from the average of all discs from the tree.

(4) $$\text{Wood}\;\text{density}\left(g/cm^{3}\right)=\text{Oven}-\text{dry}\;\text{mass}\left(g\right)/\text{Green}\;\text{volume}\;\left(cm^{3}\right)$$

### Biomass estimation

Biomass of stem bark (dry weight) was estimated via drying of bark weight taken from the disc (Eq. 5).

(5) Dryweightofstembark(kg/tree)=stembark−to−stemwoodratio×stemweight

Where:

$\text{Stem}\;\text{bark}:\text{stem}\;\text{wood}\;\text{ratio}\;\left(\%\right)=\frac{\text{Od}\;\text{wt}\;\text{of}\;\text{stem}\;\text{bark}}{\text{Od}\;\text{wt}\;\text{of}\;\text{stem}\;\text{bark}\;+\;\text{Od}\;\text{wt}\;\text{of}\;\text{stem}\;\text{wood}}$

Eq. 6 was used to estimate sawdust lost cutting each disc. This weight was added to the final weight of stem for determination of stem biomass:

(6) $$M_{\text{sd}}=\text{WD}\;\times V_{\text{sd}}$$

Where: M_sd_ is the mass of sawdust (kg); WD is wood density of each disc (kg/m^3^): V_sd_ is volume of sawdust (m^3^).

V_sd_ =0.016 (π × D^2^)/4. Where: D is diameter of each disc (m); 0.016 is the width of sawcut

Dry weight biomass of sampled trees was estimated with different component: (1) stem wood; (2) stem bark; (3) sawdust; (4) stem (1+2+3); (5) large branches; (6) small branches and foliage; (7) crown (5+6); (8) aboveground biomass (4+7); (9) rootball; (10) medium roots; (11) belowground biomass (9+10); and (12) whole tree (8+11).

Biomass can then be converted to a carbon content using conversion factors (CFs). CFs for woody trees are divided into two DBH groups: CF = 0.46 if DBH < 10 cm; CF = 0.49 if DBH ≥ 10 cm [27]. Alternatively, carbon content of the various components can be determined through additional laboratory analysis.

Allometric functions have been commonly developed using two basic measures of tree size, DBH and H [1,^10^,[12], [13], [14], [15], [16], [17], [18], [19], [20], [21]. However, using wood density in allometric models might improve model reliability over DBH alone, given that density varies among different species and with age [1]. Other studies also indicated that adding wood density (WD) and crown diameter (CD) as a variable helped to increase R^2^ for allometry [13,^14^,16]. Tree variables, including DBH, H, WD, CD or CA can be used to develop regression equations to estimate biomass and carbon accumulation of the above and belowground the components of tree. This study will use relevant variables to test and select optimal models for estimating AGB and BGB of plantation grown eucalypts.

## Additional information

Root excavation is time-consuming and costly, and it is difficult to obtain accurate samples for large trees which often have deep and widespread roots [28]. For this reason, studies involving destructive measurement methods to develop allometric equations of BGB, particularly the large roots and rootball is more limited in the literature than for AGB. Most studies used the Biomass Expansion Factor (BEF) which was developed by IPCC (2006) [2] to estimate BGB. However, indirect estimates often result in high mean absolute prediction error which ranges from 15-39% for individual species models and about 13% for stand models based on plant functional types in Australia [29]. In contrast, BGB allometric equations constructed based on a relationship between biomass and DBH, result in lower prediction errors of between 2-7% [29]. This will be more fully explained in a subsequent paper.

## Declaration of Competing Interest

The authors confirm that there are no conflicts of interest.
